# How has child growth around adiposity rebound altered in Scotland since 1990 and what are the risk factors for weight gain using the Growing Up in Scotland birth cohort 1?

**DOI:** 10.1186/s12889-016-3752-z

**Published:** 2016-10-13

**Authors:** Lawrence Doi, Andrew James Williams, John Frank

**Affiliations:** 1 Scottish Collaboration for Public Health Research and Policy, Usher Institute of Population Health Sciences and Informatics, University of Edinburgh, 20 West Richmond Street, Edinburgh, EH8 9DX UK; 2 Farr Institute at Scotland, University of Edinburgh, Nine, Edinburgh BioQuarter, 9 Little France Road, Edinburgh, EH16 4UX UK; 3 European Centre for Environment and Human Health, University of Exeter Medical School, Knowledge Spa, Royal Cornwall Hospital, Truro, Cornwall TR1 3HD UK

**Keywords:** Child, Overweight, Obesity, Adiposity rebound, Body mass index, Growth, Risk factors, Scotland, Percentile

## Abstract

**Background:**

Adiposity rebound is considered critical to the development of overweight and obesity. The purpose of this study was to investigate how growth has changed in comparison to the UK 1990 BMI growth reference curves between the ages 4–8 years and identify any marked deviations in growth. We also examined potential maternal and child risk/protective factors associated with the altered growth patterns.

**Methods:**

We used data from birth cohort 1 of the Growing Up in Scotland study. Height and weight data (*N* = 2 857) were available when the children were aged approximately 4 (sweep 4), 6 (sweep 6) and 8 years (sweep 7). For each child, percentile change per month was calculated to identify deviations from the UK 1990 growth patterns. Marked changes (>10 % annual change) in percentiles or weight category between each sweep for each child were considered as reflecting a decreasing (leptogenic), increasing (obesogenic) or no change pattern. Logistic regression was used to explore which maternal or child risk factors were associated with belonging to the different growth patterns.

**Results:**

Sixty six percent (66 %) of the cohort did not show marked changes in BMI percentile and growth compared to the UK 1990 reference population. However, the median BMI percentile of this group was around the 70th. The most common deviation in BMI percentile was early decrease (11.5 %). In terms of weight categories, contemporary maternal obesity (odd ratio (OR) =2.89; 95 % confidence interval (CI) 2.09, 3.98) and mother smoking during pregnancy (OR =1.56; 95 % CI 1.13, 2.15) were found to be significantly associated with increased odds of obesogenic growth trajectory relative to no change trajectory. Breastfeeding (OR = 1.18; 95 % CI 0.88, 1.57) was also associated with increased odds of obesogenic growth but this was not significant in the adjusted model.

**Conclusions:**

This study has shown that there is a substantial shift in the general population distribution of BMI since 1990. We identified maternal weight status as the strongest obesogenic factor and this is an indication that more innovative obesity preventive strategies should also consider intergenerational approaches.

## Background

Children’s weight is an important marker of their general development and physical health. Obese children can experience hypertension, glucose intolerance, dyslipidaemia, psychological co-morbidities, childhood asthma, chronic inflammation and obstructive sleep apnoea syndrome [1–4]. Often this unhealthy weight continues into adulthood, leading to increased risk of many negative health outcomes, including diabetes and cardiovascular diseases [2, 5, 6]. Despite the growing concern about the consequences of childhood obesity, the prevalence still remains high in many countries [7]. For example, data from Scotland show that the prevalence of childhood obesity among children aged 2 to 15, increased from 14.3 to 16.6 % between 1998 and 2008 [8].

Growth in stature and weight are not linear during childhood. For the first year of life children gain weight rapidly but then for the next four to five years their body mass index (BMI) reduces [9]. The culmination of this reduction is known as the adiposity rebound [9]. Following the adiposity rebound growth becomes more linear into adulthood [9]. Consequently, in order to monitor child growth and overweight and obesity it is necessary to compare to a reference population, which in the UK is referred to as the UK1990 [9, 10]. At the time that the UK BMI reference charts were developed, adiposity rebound was observed to happen among those of average BMI around 5–6 years of age [9, 10]. However, the rebound happened earlier (around 4.5 years for a male on the 99.6^th^ percentile) in those at higher BMI percentiles and later (around 7.5 years for a male or female on the 0.4^th^ percentile) in those at lower BMI percentiles [9]. Consequently, the period of adiposity rebound has long been considered critical to the development of overweight and obesity [9, 11]. The use of historical reference populations to compute age and sex standardised body mass index standard deviation scores (BMI-SDS also known as BMI z-scores) means that it is possible to assess the nature of the evolution of the obesity epidemic [12, 13]. In two studies Johnson et al. have explored the changing pattern and distribution of child growth, identifying that the epidemic dates back to the 1970s in the USA and demonstrating that in the UK overweight and obesity are developing earlier in childhood [12, 13].

Furthermore, having identified sub-populations whose growth patterns deviate from those observed in 1990 it becomes possible to explore potential obesogenic or leptogenic factors contributing to the different growth patterns. For example, a recent systematic review of nine studies demonstrated that key risk factors for accelerated child growth were primiparity, maternal smoking during pregnancy, lower birth weight, and early weaning [14]. Using data from the Generation 1 study in Australia, Giles et al. [15] found that maternal obesity in early pregnancy was the most important risk factor among children in the accelerating growth trajectory group as compared to those in the intermediate growth trajectory group (Odds Ratio (OR) 3.72; 95 % CI 1.15–12.05). Understanding the changes in child growth and the determinants of the changes offers immense potential for early obesity prevention. For instance, more innovative programmes can be tailored to preventable and remediable risk factors identified among such susceptible groups of children.

Consequently, using Growing Up in Scotland (GUS) [16], a large nationally representative cohort study of Scottish mothers and children, this study had two aims. The first aim was to identify children who have markedly deviated from the growth trajectory observed in 1990, particularly related to the timing of adiposity rebound (ages 4–8 years). The second aim was to examine potential maternal and child risk/protective factors for the altered growth patterns, culminating in consideration of how the findings may inform future intervention strategies.

## Methods

### Study population

Growing Up in Scotland is a nationally representative cohort study aimed at tracking the lives of three cohorts of Scottish children from the early years, through childhood and beyond. The full design and methods of the Growing Up in Scotland study have been described elsewhere [16]. This study was based on birth cohort 1 which began with newborns in 2004/05 followed-up annually until they were 6 years old (2010/11) after which follow-up reduced to biennial. Only one child per household was enrolled in GUS, where multiple children were eligible (e.g. twins) one was randomly chosen. Direct, in-the-home measurements of weight and height on the children were available for the sweeps in 2008/09, 2010/11 and 2012/13 when the children were on average aged approximately 4, 6 and 8 years respectively during which adiposity rebound would be expected to have occurred.

**Fig. 1:**
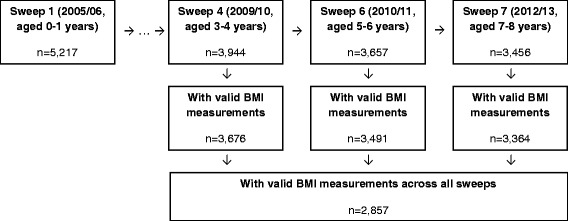
Study sample size

As part of the Growing Up in Scotland study a broad range of child and maternal variables are collected. The key domains covered by data collection are: cognitive, social, emotional and behavioural development; physical and mental health and wellbeing; childcare, education and employment; home, family, community and social networks; and involvement in offending and risky behaviour [16]. A number of variables from these domains have either been empirically or theoretically identified as obesogenic or leptogenic factors; those explored in this study are listed in Table 1 [14, 17–19]. Given the focus of study on adiposity rebound, height and weight data at a minimum of three time points would be required to identify the rebound and hence it was necessary to conduct a “complete case” analysis. Fig. 1 illustrates the flow of data leading to the final sample size.

**Table 1:** Explanatory variables and their definitions

Variable	Sweep (Age of child) when data was collected	Definition	Sample
Gender	Sweep 1 (10 months)	Male	49.6 %
		Female	50.4 %
Number of children in family	Sweep 7 (7–8 years)	One	14.6 %
		Two	54.0 %
		Three	24.2 %
		Four or more	7.2 %
Ethnicity	Sweep 1 (10 months)	White	97.6 %
		Non-white	2.4 %
Scottish Index of Multiple Deprivation (SIMD)	Sweep 7 (7–8 years)	Q1 (Least deprived)	25.2 %
		Q2	24.4 %
		Q3	21.6 %
		Q4	15.7 %
		Q5 (Most deprived)	13.1 %
Maternal weight status	Sweep 6 (5–6 years)	Healthy/underweight (BMI <25 kg/m^2^)	44.9 %
		Overweight (≥25 kg/m^2^ to >30 kg/m^2^)	31.5 %
		Obese (≥30 kg/m^2^)	23.6 %
Mother smoked in pregnancy	Sweep 1 (10 months)	No	81.6 %
		Yes	18.4 %
If child ever breastfed	Sweep 1 (10 months)	Yes	69.8 %
		No	30.2 %
Birth weight	Sweep 1 (10 months)	Normal (>2.5 kg)	94.2 %
		Low (<2.5 kg)	5.8 %
Total sample size			2,278

### Methods

Cole et al. [20] advise that the best measures for assessing changes in child growth are either BMI or BMI percentile. Therefore, given the aim of this study to make comparisons with the 1990 data each BMI measurement was converted into a percentile of the UK1990 reference population [9]. Those measurements which equated to a BMI-SDS greater than ±5 (*n* = 5) were considered invalid and subsequently not included in the analysis. Each measurement was categorised as underweight, healthy weight, overweight or obese using 2^nd^, 85^th^ and 95^th^ percentiles as cut-points. Previous research by one of the investigators had identified merits of treating overweight and obesity as mutually exclusive categories rather than considering all obese to also be overweight and subsequently this classification has been used in this study (overweight 85^th^- < 95^th^ centile, obese ≥95^th^ centile) [21].

Next, the change in weight category and percentile between each sweep for each individual was calculated. To account for the fact that the measurement in each sweep had not been taken at precisely the same age, the change in percentile was divided by the change in age (in months), to give a percentile change per month. The method derived by Cole [22] means that a child on the 50^th^ centile has the median BMI for a child of their gender and age in 1990; therefore a child who remains on approximately the same percentile between sweeps is following the growth pattern observed in 1990 including the adiposity rebound. As well as exploring ‘phase shift’ changes in weight category, marked changes in percentile were evaluated. The authors considered that an annual change of more than 10 percentiles would be considered a marked change (monthly change ≥ ±0.84 percentiles). This was an arbitrary decision. However, the authors clinical experience suggests that clinical monitoring of child weight using growth charts or a computer system is likely to highlight >10 percentile change across a year and draw the attention of relevant individuals. Consequently, changes in weight category and percentile were categorised as reflecting either a decrease, increase or no change between sweeps.

Two separate analyses were then undertaken to address the second aim, examining either BMI percentile or weight category changes. Those remaining in the healthy weight category, or in the healthy weight category and not demonstrating any marked change in percentile were considered the reference category. Those moving from underweight, healthy weight, or overweight to overweight or obese or showing marked increases in percentile were considered to be experiencing obesogenic factors. While those moving from obese or overweight to overweight or healthy weight or showing marked decreases in percentile (excluding those becoming underweight) were considered to be experiencing leptogenic factors. The low prevalence of underweight meant that movements into this category were not evaluated. Dummy variables were derived for the obesogenic and leptogenic patterns comparing them with the reference category; logistic regression (single and multivariable) was used to explore which of the variables in Table 1 were associated with belonging to the different growth patterns. Five of the variables in Table 1 are time invariant; gender, ethnicity, mother smoked during pregnancy, breastfed and birth weight. For the other three variables, the most recent data were used: number of children in the family, Scottish Index of Multiple Deprivation and mother’s weight status. All analyses were undertaken in Stata 14 [23] and were two-tailed with *ɑ* = 0.05. All the variables in Table 1 were included in each of the final models (i.e. no model fitting was undertaken to avoid the risk of overfitting). Although longitudinal weightings are provided for the Growing Up in Scotland study, these were not used as the complete case approach meant that results would not be representative of the whole cohort in any case, and therefore could not be considered representative of all Scottish children.

## Results

Presented in Table 2 are the cross-sectional ages, BMI percentiles and weight categories of the complete cases (*n* = 2,857). On average the ages of the children in each of the sweeps were; sweep 4, 3 years and 10 months, sweep 6, 5 years and 10 months, and sweep 7, 7 years and 10 months. The BMI percentiles were positively skewed with interquartile range around 40 percentiles, and the median between the 60th and 70th percentiles.

**Table 2:** Sample characteristics (*n* = 2,857)

		Mean	Standard deviation	Median	Interquartile range
Age (months)	Sweep 4	46.16	0.46	46.00	46.00 to 46.00
	Sweep 6	70.21	0.46	70.00	70.00 to 70.00
	Sweep 7	94.39	0.73	94.00	94.00 to 95.00
BMI percentile	Sweep 4	63.64	26.49	68.39	43.28 to 86.65
	Sweep 6	60.00	27.24	63.16	39.50 to 83.57
	Sweep 7	59.74	28.22	63.16	36.72 to 84.84
Weight category		Underweight	Healthy weight	Overweight	Obese
	Sweep 4	14 (0.49 %)	2,066 (72.31 %)	469 (16.42 %)	308 (10.78 %)
	Sweep 6	25 (0.88 %)	2,172 (76.02 %)	381 (13.34 %)	279 (9.77 %)
	Sweep 7	19 (0.67 %)	2,130 (74.55 %)	352 (12.32 %)	356 (12.46 %)

Considering changes in weight category, 67.4 % (*n* = 1,925) of the cohort did not change category between sweeps. Of those who did not change 89.7 % were healthy weight, 3.4 % overweight and 6.8 % obese, less than 1 % were underweight. The most common deviation in growth was an early (between sweeps 4 and 6) decrease (9.6 %) in category, followed by a late increase (6.2 %) and early increase (4.6 %). Only around 2 % (*n* = 64) of the cohort showed a continuous increase or decrease in weight category, while 185 switched back and forth between categories across the three sweeps. For those who were underweight in sweep 4 the most common phase shift was an early shift to healthy weight (50.0 %), for those of healthy weight it was most common for them to remain healthy weight (83.5 %) across all sweeps; many of those overweight early shifted to healthy weight (42.2 %); and for those obese, 42.5 % remained obese. When considering marked changes in BMI percentile (≥ ± 10 percentiles per year) 66.1 % of the cohort did not show marked changes in BMI percentile. The majority of these were healthy weight (69.2 %), less than 1 % was underweight, 14.2 % were overweight and 16.0 % were obese in sweep 7. The median (interquartile range) BMI percentile of the no change group was 73.1 (44.1 to 89.7) in sweep 4, 71.1 (42.1 to 87.9) in sweep 6 and 68.6 (40.9 to 88.8) in sweep 7. The most common deviations in growth in terms of marked changes in BMI percentile were: early decrease (11.5 %), late decrease (6.9 %), late increase (5.2 %) and early increase (5.1 %). Only 1.5 % demonstrated a consistent marked increases or decreases in BMI percentile, while 111 switched back and forth between marked increases and decreases.

The adjusted and unadjusted results of the logistic regression exploring the risk factors for obesogenic or leptogenic growth patterns in terms of weight categories and percentiles are reported in Tables 3 and 4. More factors were found to be associated with changes in weight category than in BMI percentile. Females were more likely to be in the obesogenic group compared to the leptogenic in terms of both weight categories and percentiles. Although rarely statistically significant (power was limited by the small proportion of the Scottish population not born in the UK), it appears that those of White ethnicity are more likely to deviate from the UK1990 growth patterns. Family size and birth weight were not found to be statistically significantly associated with altered growth. Contemporary maternal weight status, mother smoked during pregnancy and breastfeeding were found to be most markedly associated with increased odds of altered growth. Although, socioeconomic status is often found to be associated with weight status, it was not a significant variable in any of the adjusted models. However, maternal weight status, smoking and breastfeeding are all correlated with socioeconomic status. Contemporary maternal weight status was the most consistent factor in obesogenic growth.

**Table 3:** Logistic regression of potential obesogenic and leptogenic child and maternal variables based on weight categories (UK1990), odds ratios (95 % confidence interval)

Factors	Obesogenic vs. no change (*n* = 1,806)		Leptogenic vs. no change (*n* = 1,860)		Obesogenic vs. leptogenic (*n* = 600)	
	Unadjusted	Adjusted	Unadjusted	Adjusted	Unadjusted	Adjusted
Gender						
Male	1	1	1*	1*	1*	1*
Female	0.98 (0.76 to 1.27)	0.94 (0.72 to 1.23)	0.66 (0.52 to 0.84)	0.67 (0.53 to 0.86)	1.48 (1.07 to 2.04)	1.49 (1.06 to 2.09)
Number of children in family						
One	1	1	1	1	1	1
Two	0.86 (0.60 to 1.25)	0.95 (0.65 to 1.38)	1.14 (0.79 to 1.65)	1.15 (0.79 to 1.68)	0.76 (0.47 to 1.22)	0.79 (0.48 to 1.30)
Three	0.89 (0.59 to 1.35)	1.01 (0.65 to 1.55)	1.24 (0.82 to 1.85)	1.26 (0.84 to 1.91)	0.72 (0.42 to 1.23)	0.74 (0.42 to 1.29)
Four or more	0.77 (0.43 to 1.37)	0.72 (0.40 to 1.31)	0.68 (0.36 to 1.25)	0.70 (0.38 to 1.31)	1.14 (0.52 to 2.49)	1.14 (0.50 to 2.57)
Ethnicity						
White	1*	1	1*	1*	1	1
Other	0.12 (0.02 to 0.91)	0.14 (0.02 to 1.07)	0.10 (0.01 to 0.76)	0.11 (0.01 to 0.79)	1.20 (0.07 to 19.25)	1.49 (0.09 to 24.72)
Scottish Index of Multiple Deprivation (SIMD)						
Least deprived (Q1)	1*	1	1	1	1	1
Q2	1.19 (0.81 to 1.76)	1.01 (0.67 to 1.50)	1.07 (0.77 to 1.50)	1.05 (0.75 to 1.47)	1.11 (0.69 to 1.78)	1.12 (0.69 to 1.83)
Q3	1.22 (0.82 to 1.82)	0.94 (0.62 to 1.42)	0.94 (0.66 to 1.33)	0.90 (0.63 to 1.30)	1.30 (0.80 to 2.13)	1.13 (0.68 to 1.87)
Q4	1.53 (1.01 to 2.31)	1.16 (0.75 to 1.80)	0.88 (0.59 to 1.30)	0.91 (0.60 to 1.37)	1.75 (1.03 to 2.96)	1.32 (0.76 to 2.30)
Most deprived (Q5)	2.11 (1.39 to 3.21)	1.49 (0.95 to 2.34)	1.30 (0.87 to 1.92)	1.29 (0.85 to 1.98)	1.63 (0.97 to 2.74)	1.21 (0.69 to 2.11)
Maternal weight status (at Sweep 6)						
Healthy weight	1*	1*	1	1*	1*	1*
Overweight	1.40 (1.01 to 1.93)	1.43 (1.04 to 1.99)	1.30 (0.99 to 1.71)	1.35 (1.03 to 1.79)	1.08 (0.73 to 1.59)	1.04 (0.70 to 1.56)
Obese	3.00 (2.20 to 4.11)	2.89 (2.09 to 3.98)	1.40 (1.03 to 1.92)	1.48 (1.07 to 2.04)	2.14 (1.44 to 3.19)	2.10 (1.39 to 3.16)
Mother smoked in pregnancy						
No	1*	1*	1	1*	1	1
Yes	1.76 (1.30 to 2.38)	1.56 (1.13 to 2.15)	1.32 (0.98 to 1.77)	1.43 (1.04 to 1.98)	1.34 (0.92 to 1.96)	1.17 (0.78 to 1.76)
If child was ever breastfed						
Yes	1*	1	1	1	1*	1*
No	1.53 (1.17 to 2.00)	1.18 (0.88 to 1.57)	0.89 (0.68 to 1.16)	0.78 (0.58 to 1.04)	1.72 (1.22 to 2.44)	1.55 (1.07 to 2.24)
Birth weight						
Normal	1	1	1	1	1	1
Low	1.06 (0.63 to 1.78)	0.93 (0.54 to 1.60)	0.67 (0.38 to 1.19)	0.66 (0.36 to 1.18)	1.58 (0.77 to 3.23)	1.37 (0.65 to 2.90)

*p < 0.05, Q = quintile

No change defined as those remaining in the healthy weight category across the three sweeps. Obesogenic defined as those moving from underweight, healthy weight, or overweight to overweight or obese across the three sweeps. Leptogenic defined as those moving from obese or overweight to overweight or healthy weight across the three sweeps

**Table 4:** Logistic regression of potential obesogenic and leptogenic child and maternal variables based on marked changed in body mass index percentile (≥ ± 10 percentiles per year), odds ratios (95 % confidence interval)

Factors	Obesogenic vs. no change (*n* = 1,375)		Leptogenic vs. no change (*n* = 1,568)		Obesogenic vs. leptogenic (*n* = 739)	
	Unadjusted	Adjusted	Unadjusted	Adjusted	Unadjusted	Adjusted
Gender						
Male	1	1	1*	1	1*	1*
Female	1.14 (0.87 to 1.49)	1.08 (0.82 to 1.42)	0.79 (0.94 to 0.99)	0.81 (0.65 to 1.00)	1.43 (1.06 to 1.94)	1.43 (1.04 to 1.95)
Number of children in family						
One	1	1	1	1	1	1
Two	0.76 (0.52 to 1.11)	0.82 (0.55 to 1.20)	0.90 (0.65 to 1.25)	0.88 (0.63 to 1.23)	0.85 (0.55 to 1.31)	0.90 (0.58 to 1.41)
Three	0.72 (0.46 to 1.13)	0.79 (0.51 to 1.25)	1.24 (0.86 to 1.77)	1.22 (0.85 to 1.75)	0.59 (0.36 to 0.96)	0.65 (0.39 to 1.09)
Four or more	1.12 (0.64 to 1.96)	1.12 (0.63 to 1.99)	1.18 (0.72 to 1.91)	1.19 (0.73 to 1.95)	0.95 (0.51 to 1.79)	0.97 (0.51 to 1.87)
Ethnicity						
White	1	1	1	1	1	1
Other	0.44 (0.13 to 1.45)	0.43 (0.13 to 1.47)	0.82 (0.39 to 1.70)	0.72 (0.34 to 1.51)	0.53 (0.15 to 1.96)	0.55 (0.14 to 2.11)
Scottish Index of Multiple Deprivation (SIMD)						
Least deprived (Q1)	1	1	1	1	1	1
Q2	1.14 (0.77 to 1.68)	1.06 (0.71 to 1.58)	0.99 (0.73 to 1.34)	0.98 (0.72 to 1.34)	1.15 (0.74 to 1.78)	1.02 (0.65 to 1.60)
Q3	1.02 (0.68 to 1.53)	0.93 (0.61 to 1.40)	0.90 (0.66 to 1.23)	0.92 (0.67 to 1.27)	1.13 (0.78 to 1.78)	0.94 (0.58 to 1.51)
Q4	1.26 (0.82 to 1.95)	1.08 (0.69 to 1.70)	0.99 (0.70 to 1.40)	1.04 (0.73 to 1.49)	1.28 (0.79 to 2.07)	1.04 (0.62 to 1.73)
Most deprived (Q5)	1.76 (1.13 to 2.74)	1.43 (0.89 to 2.30)	1.15 (0.79 to 1.66)	1.23 (0.83 to 1.84)	1.54 (0.94 to 2.51)	1.13 (0.67 to 1.92)
Maternal weight status (at Sweep 6)						
Healthy weight	1*	1*	1	1	1*	1*
Overweight	0.99 (0.72 to 1.38)	0.99 (0.72 to 1.38)	1.08 (0.85 to 1.38)	1.09 (0.85 to 1.39)	0.92 (0.64 to 1.31)	0.92 (0.63 to 1.32)
Obese	1.66 (1.20 to 2.31)	1.58 (1.13 to 2.20)	0.89 (0.67 to 1.20)	0.91 (0.67 to 1.23)	1.86 (1.27 to 2.71)	1.78 (1.20 to 2.64)
Mother smoked in pregnancy						
No	1*	1	1	1	1*	1
Yes	1.48 (1.07 to 2.05)	1.32 (0.93 to 1.87)	0.89 (0.66 to 1.19)	0.87 (0.64 to 1.19)	1.66 (1.14 to 2.43)	1.48 (0.99 to 2.22)
If child was ever breastfed						
Yes	1	1	1	1	1	1
No	1.17 (0.88 to 1.55)	0.97 (0.71 to 1.33)	0.91 (0.71 to 1.15)	0.90 (0.70 to 1.16)	1.29 (0.93 to 1.78)	1.11 (0.78 to 1.56)
Birth weight						
Normal	1	1	1	1	1	1
Low	1.35 (0.82 to 2.22)	1.21 (0.73 to 2.03)	0.79 (0.49 to 1.27)	0.82 (0.51 to 1.34)	1.71 (0.94 to 3.11)	1.43 (0.76 to 2.67)

*p < 0.05, Q = quintile

No change defined as those in the healthy weight category in sweep 4 and not demonstrating any marked change in percentile across the three sweeps. Obesogenic defined as those in the healthy weight, or overweight categories in sweep 4 showing marked increases in percentile across the three sweeps. Leptogenic defined as those in the obese or overweight categories in sweep 4 showing marked decreases in percentile (excluding those becoming underweight) across the three sweeps

Only in terms of weight categories were those who were not breastfed more likely to be increasing compared to be decreasing category (Table 3). Notably, although this was not significant in the models related to BMI percentile (Table 4), in those related to weight category contemporary maternal weight status (particularly obesity) and history of smoking during the pregnancy were associated with higher odds of being in both the obesogenic or leptogenic groups, compared to the no change group. These associations particularly in relation to smoking were of similar orders of magnitude (Table 3). This finding was substantiated in the model comparing the obesogenic and leptogenic groups where smoking during pregnancy was not found to significantly differ between the groups; however maternal weight status was statistically significantly associated with obesogenic growth.

## Discussion

Three sweeps of data (*n* = 2,857) from birth cohort 1 of the Growing Up in Scotland cohort study have been analysed to explore how child growth (in terms of stature and weight) around the age of adiposity rebound (3–8 years of age) has changed since the UK1990 growth standard was defined [9]. Two-thirds of the sample were found to be demonstrating growth which did not markedly deviate from the patterns seen in 1990 [9]. However, the median BMI percentile of this group was around 70^th^ percentile in the 1990 data, which demonstrates a major population shift in the distribution of BMI since 1990. Those who were underweight, healthy weight or overweight in the first sweep analysed (3–4 years old) were most likely to become or remain healthy weight by the final sweep (7–8 years old). However, those who were obese in the first sweep were most likely to remain obese, which would suggest that although the adiposity rebound is a time of altering growth, efforts to prevent obesity need to begin before the adiposity rebound and therefore before mandatory education. In fact, among the study sample of those who showed marked deviations from the UK1990 growth patterns, reductions in weight category or BMI percentile were more common. Contemporary maternal weight status was the strongest obesogenic factor identified. However, *in utero* or early life factors such as mother smoking during pregnancy and not being breastfeed were also found to be statistically significant in some models.

In their study of five UK cohorts Johnson et al. [12] found that the onset of overweight and obesity had been starting earlier in life. The finding within GUS that 10.8 % of the sample were already obese by around 3–4 years of age clearly demonstrates the early onset of much child obesity. Furthermore, we found that 42.5 % of that 10.8 % remained obese up to 7–8 years of age. These children may have developed more intransigent obesity, but to be sure it would obviously be necessary to follow them up in later life, and to examine their risks for morbidity and mortality to see how they compare with risks for those who develop obesity in later life. Otherwise, the analysis clearly indicates that 3–8 years of age is a time of healthy changes in weight, in that 71.4 % of the underweight children and 55 % of the overweight children developed healthy weight. Johnson et al. [13] in analysing the Fels Longitudinal Study, found that during the obesity epidemic the pattern seemed to be lower weight prior to the adiposity rebound and subsequent rapid growth. This observation may support the idea that the two patterns of obesity – obesity developing before and after adiposity rebound – are different, and could be the result of different influences (e.g. in terms of genetics and environment).

Our finding that maternal weight status is an important obesogenic (and possibly leptogenic) factor is consistent with a number of studies. This association remains irrespective of when maternal weight was measured [24]. For example, other studies have found that maternal overweight or obesity status pre-pregnancy [19, 25], early pregnancy [17], at 1.5 years [26, 27] or when the child is aged 3 years [19], were all associated with childhood weight gain. Toschke et al. [24] found that maternal weight status was associated with both a positive shifting and skewing in the BMI distribution. Further investigation is, however, needed to clarify the differential contribution to childhood obesity of this risk factor in terms of genetic and environmental components. Nevertheless, the influence of maternal overweight and obesity status on children’s weight gain is an important pointer for childhood obesity preventative efforts.

As in our findings, the role of smoking during pregnancy and breastfeeding is not yet conclusive in the literature. Ong et al. [28] found no association between smoking and child weight status at age 5 years, several studies have found this factor to be an important predictor of weight gain in children [19, 24, 27, 29, 30]. Nevertheless, aside from the evidence of the effect of prenatal smoking on growth catch-up, particularly during the first 12 months of life [14], there is some evidence that the effect on childhood overweight or obesity can particularly manifest in children after 7 years [31]. Although the underlying mechanism is unknown, our findings suggest that the effect of prenatal smoking is closely entangled with deprivation (all smoking is strongly associated with deprivation in Scotland). Evidence suggests that breastfeeding anytime in the first 12 months of life is a protective factor for childhood overweight [32]. We found that never having been breastfed was an obesogenic factor. This is similar to the findings of Li et al. [25] and Toschke et al. [24] even though differences often exist across studies in how this variable is defined and measured. However, it is unclear whether this effect represents a long-term effect of breastfeeding or the fact that breastfeeding is correlated with deprivation [33].

Exploring child growth, rather than cross sectional weight status, is a significant strength of this study, and a virtue of using the Growing Up in Scotland cohort. Even though the complete case analysis means that the results of this study cannot be considered representative of Scotland like the full cohort. However, our weight status outcome was based on direct measurements rather than reported measures of the child’s weight and height. Unfortunately, the maternal weight status data in GUS are self-reported. Several important potential risk factors for childhood obesity, such as physical activity and dietary information were not included in the multivariable analysis. Although this might be a limitation, these variables are subjectively reported in GUS and are not available longitudinally. For the purpose of the paper BMI-SDS greater than ±5 was considered an invalid BMI. Only five participants were excluded by this approach. These five participants were already excluded from the study sample size of 2,857 and are not additional exclusions. The decision to use annual change in BMI greater than 10 percentiles as indicating a marked change was arbitrary. Ford et al. [34] and Kolgaard et al. [35, 36] have found that changes in BMI-SDS of around 0.25 are associated with clinically relevant changes. Within the age groups studied a 10 percentile change across a year equates to at least a change in BMI-SDS of 0.25. However, other specifications of marked changes in BMI percentile could be examined in future research.

## Conclusions

The authors believe this to be the first study to attempt to distinguish between the overall population shift in BMI and the increasingly positive skew and the findings support a number of recommendations for practice and research. It is crucial for research to explore growth rather than cross-sectional weight, especially when it is possible to compare growth with previous cohorts and identify high- or low-risk subpopulations. The findings also inform the selection of which of the two approaches to prevention proposed by Geoffrey Rose might be more appropriate for obesity – should childhood obesity preventative measures adopt a population-wide or high-risk approach [37]? Any targeted (high-risk approach) childhood obesity preventative strategies should not overlook risk factors such as maternal overweight or obesity, smoking during pregnancy and possibly breastfeeding. However, the multiple determinants of these risk factors are themselves complex and may require a population-wide approach. Our results support the need for whole family interventions which start early and further suggest that primary school interventions may be too late. Although the rapid weight gain following adiposity rebound found by Johnson et al. [13] may support the need for primary school intervention. While adopting the high-risk approach seems more pragmatic and economically attractive, there appears to have been a marked (~20 percentile point) shift in the general population distribution of BMI, which indicates the need for population-wide approaches.

## Abbreviations

95 % CI: 95 % confidence interval
BMI: Body mass index
BMI-SDS: Body mass index standard deviation score
GUS: Growing Up in Scotland study
OR: Odds ratio
UK1990: United Kingdom 1990 BMI growth reference

## References

[1] Strauss RS (2000). Childhood obesity and self-esteem. Pediatrics.

[2] Reilly JJ, Methven E, McDowell ZC, Hacking B, Alexander D, Stewart L, Kelnar CJ (2003). Health consequences of obesity. Arch Dis Child.

[3] Reilly JJ (2005). Descriptive epidemiology and health consequences of childhood obesity. Best Pract Res Clin Endocrinol Metab.

[4] Weiss R, Caprio S (2005). The metabolic consequences of childhood obesity. Best Pract Res Clin Endocrinol Metab.

[5] Singh AS, Mulder C, Twisk JW, van Mechelen W, Chinapaw MJ (2008). Tracking of childhood overweight into adulthood: a systematic review of the literature. Obes Rev.

[6] Freedman DS, Khan LK, Dietz WH, Srinivasan SR, Berenson GS (2001). Relationship of childhood obesity to coronary heart disease risk factors in adulthood: The Bogalusa Heart Study. Pediatrics.

[7] Wang Y, Lobstein T (2006). Worldwide trends in childhood overweight and obesity. Int J Pediatr Obes.

[8] Gray L, Leyland AH, Rutherford L, Hinchliffe S, Sharp C (2012). Obesity. Scottish Health Survey: main report, Vol 1.

[9] Cole TJ, Freeman JV, Preece MA (1995). Body mass index reference curves for the UK, 1990. Arch Dis Child.

[10] Freeman JV, Cole TJ, Chinn S, Jones PRM, White EM, Preece MA (1995). Cross-sectional stature and weight reference curves for the UK 1990. Arch Dis Child.

[11] Dietz WH (1997). Periods of risk in childhood for the development of adult obesity - what do we need to learn?. J Nutr.

[12] Johnson W, Li L, Kuh D, Hardy R (2015). How has the age-related process of overweight or obesity development changed over time? Co-ordinated analyses of individual participant data from five United Kingdom birth cohorts. PLoS Med.

[13] Johnson W, Soloway LE, Erickson D, Choh AC, Lee M, Chumlea WC, Siervogel RM, Czerwinski SA, Towne B, Demerath EW (2012). A changing pattern of childhood BMI growth during the 20th century: 70 y of data from the Fels Longitudinal Study. Am J Clin Nutr.

[14] Chrestani MA, Santos IS, Horta BL, Dumith SC, Dode MASD (2013). Associated factors for accelerated growth in childhood: a systematic review. Matern Child Hlth J.

[15] Giles LC, Whitrow MJ, Davies MJ, Davies CE, Rumbold AR, Moore VM (2015). Growth trajectories in early childhood, their relationship with antenatal and postnatal factors, and development of obesity by age 9 years: results from an Australian birth cohort study. Int J Obes (Lond).

[16] Growing Up in Scotland: Cohort 1, Sweeps 1–6, 2005–2011 [http://dx.doi.org/10.5255/UKDA-SN-5760-4]. Accessed 28 Apr 2016.

[17] Giles LC, Whitrow MJ, Rumbold AR, Davies CE, de Stavola B, Pitcher JB, Davies MJ, Moore VM (2013). Growth in early life and the development of obesity by age 9 years: are there critical periods and a role for an early life stressor?. Int J Obes.

[18] Reilly JJ, Armstrong J, Dorosty AR, Emmett PM, Ness A, Rogers I, Steer C, Sherriff A (2005). Early life risk factors for obesity in childhood: cohort study. Brit Med J.

[19] Griffiths LJ, Hawkins SS, Cole TJ, Dezateux C, Millennium Cohort Study Child Health G (2010). Risk factors for rapid weight gain in preschool children: findings from a UK-wide prospective study. Int J Obes (Lond).

[20] Cole TJ, Faith MS, Pietrobelli A, Heo M (2005). What is the best measure of adiposity change in growing children: BMI, BMI %, BMI z-score or BMI centile?. Eur J Clin Nutr.

[21] Williams AJ, Wyatt KM, Williams CA, Logan S, Henley WE (2015). Exploring the potential of a school impact on pupil weight status: exploratory factor analysis and repeat cross-sectional study of the National Child Measurement Programme. PLoS One.

[22] Cole TJ (1990). The LMS method for constructing normalized growth standards. Eur J Clin Nutr.

[23] StataCorp (2015). Stata statistical software: release 14.

[24] Toschke AM, von Kries R, Beyerlein A, Ruckinger S (2008). Risk factors for childhood obesity: shift of the entire BMI distribution vs. shift of the upper tail only in a cross sectional study. BMC Public Health.

[25] Li C, Goran MI, Kaur H, Nollen N, Ahluwalia JS (2007). Developmental trajectories of overweight during childhood: role of early life factors. Obesity (Silver Spring).

[26] Carter MA, Dubois L, Tremblay MS, Taljaard M, Jones BL (2012). Trajectories of childhood weight gain: the relative importance of local environment versus individual social and early life factors. PLoS One.

[27] Pryor LE, Tremblay RE, Boivin M, Touchette E, Dubois L, Genolini C, Liu X, Falissard B, Cote SM (2011). Developmental trajectories of body mass index in early childhood and their risk factors: an 8-year longitudinal study. Arch Pediatr Adolesc Med.

[28] Ong KKL, Preece MA, Emmett PM, Ahmed ML, Dunger DB (2002). Size at birth and early childhood growth in relation to maternal smoking, parity and infant breast-feeding: longitudinal birth cohort study and analysis. Pediatr Res.

[29] Toschke AM, Koletzko B, Slikker W, Hermann M, von Kries R (2002). Childhood obesity is associated with maternal smoking in pregnancy. Eur J Pediatr.

[30] Suzuki K (2015). Longitudinal analyses of childhood growth: evidence from Project Koshu. J Epidemiol.

[31] Chen A, Pennell ML, Klebanoff MA, Rogan WJ, Longnecker MP (2006). Maternal smoking during pregnancy in relation to child overweight: follow-up to age 8 years. Int J Epidemiol.

[32] Weng SF, Redsell SA, Swift JA, Yang M, Glazebrook CP (2012). Systematic review and meta-analyses of risk factors for childhood overweight identifiable during infancy. Arch Dis Child.

[33] Oakley LL, Renfrew MJ, Kurinczuk JJ, Quigley MA: Factors associated with breastfeeding in England: an analysis by primary care trust. BMJ Open 2013, 3(6):e002765. doi:10.1136/bmjopen-2013-002765. 10.1136/bmjopen-2013-002765 PMC3693424 23794590

[34] Ford AL, Hunt LP, Cooper A, Shield JPH (2010). What reduction in BMI SDS is required in obese adolescents to improve body composition and cardiometabolic health?. Arch Dis Child.

[35] Kolsgaard MLP, Joner G, Brunborg C, Anderssen SA, Tonstad S, Andersen LF (2011). Reduction in BMI z-score and improvement in cardiometabolic risk factors in obese children and adolescents. The Oslo Adiposity Intervention Study - a hospital/public health nurse combined treatment. BMC Pediatr.

[36] Kolsgaard MLP, Joner G, Brunborg C, Anderssen SA, Tonstad S, Andersen LF (2012). Erratum to: “Reduction in BMI z-score and improvement in cardiometabolic risk factors in obese children and adolescents. The Oslo adiposity intervention study - a hospital/public health nurse combined treatment.”. BMC Pediatr.

[37] Rose G (2001). Sick individuals and sick populations. Int J Epidemiol.

